# Cellular and systemic modifiers of alpha-synuclein proteostasis

**DOI:** 10.1098/rstb.2025.0275

**Published:** 2026-10-01

**Authors:** Suzanne Couzijn, Anna P. Ainslie, Alejandro Herron-Bedoya, Ellen A. A. Nollen

**Affiliations:** European Research Institute for the Biology of Ageing (ERIBA), University Medical Centre Groningen (UMCG), 9713AV Groningen, GR, The Netherlands

**Keywords:** alpha-synuclein, protein condensation, protein aggregation, proteostasis, ageing

## Abstract

Ageing is a primary risk factor for neurodegenerative disorders, including Parkinson's disease (PD). As individuals age, their cells become less efficient in maintaining protein homeostasis, leading to an increased likelihood of protein misfolding and aggregation. A hallmark of PD and other synucleinopathies is the accumulation of alpha-synuclein protein aggregates in affected neurons, a process that is exacerbated by ageing. While cellular mechanisms that regulate protein aggregation have been a primary focus of research, recent studies suggest that other, systemic age-related mechanisms may contribute to alpha-synuclein toxicity. Understanding these alternative pathways is crucial for the development of effective therapeutic strategies to combat neurodegenerative diseases, such as PD. In this review, we synthesize current insights into the biological mechanisms underlying alpha-synuclein toxicity at the organismal level. We highlight key open questions and discuss how these findings may inform the development of targeted interventions to prevent or delay age-related synucleinopathies.

This article is part of the Theo Murphy meeting issue ‘ProteostaSys: a systems view of proteostasis’.

## 1. Alpha-synuclein structure and physiological role

Synucleinopathies are neurodegenerative diseases characterized by pathological changes in the alpha-synuclein protein, including condensation, aggregation and intracellular spreading [1,2]. Alpha-synuclein is a small acidic protein of 140 amino acids that can be divided into three distinct domains: the membrane-binding N-terminal region (1–60), the hydrophobic non-amyloid-component (NAC) domain (61–95) and the disordered and flexible C-terminal domain (96–140). The hydrophobic N-terminus consists of seven imperfect repeats of 11 residues (the KTKEGV motif) that can adopt an alpha-helical structure involved in lipid binding [3–5]. By contrast, the NAC domain of alpha-synuclein can form highly structured beta-sheets and this domain is, therefore, also associated with alpha-synuclein aggregation and amyloid formation [6,7]. The C-terminus is polar, negatively charged, hydrophilic, often phosphorylated and can interact with calcium and synaptic vesicles [8,9]. It was also shown that the C-terminal domain of alpha-synuclein can regulate the liquid–liquid phase separation (LLPS) of the protein, which is a process that can lead to protein condensates and inclusions [9,10]. The protein has mainly been defined as a natively unfolded protein monomer, however it has been shown that alpha-synuclein endogenously also exists as folded tetramers of about 58 kDa, which have little aggregation propensity [11,12].

The alpha-synuclein protein is encoded by the alpha-synuclein gene (*SNCA*) that is mainly expressed in different neuronal populations of both the peripheral and the central nervous system, and is mostly localized at the presynaptic terminals in the brain [13,14]. However, it was also found to be expressed in other cell types, including erythrocytes, platelets and other non-neuronal cells, such as muscle, liver, kidney, heart and lung tissue [2,15,16]. Most currently identified point mutations in the *SNCA* gene are located at the membrane-binding domain of the N-terminus. These mutations have been associated with different diseases, such as Parkinson's disease (PD) or dementia with Lewy Bodies (DLB) (these are: A18T, A29S, A30P/G, E46K, H50Q, G51D, A53E/T/V, T72M and E83Q) [17–20]. Furthermore, large gene copy number variations that result in duplications or triplications of the *SNCA* gene also lead to disease [21–23]. Furthermore, alpha-synuclein can undergo post-translational modifications (PTMs), including but not limited to phosphorylation, acetylation, ubiquitination, SUMOylation, glycosylation, nitration and methylation. [24–26]. Additionally, truncations, but most notably C-terminal truncations, also affect the aggregation propensity of alpha-synuclein [27,28]. Here, loss of the acidic tail of the protein increases the tendency of the protein to undergo polymerization into beta-sheet-rich fibrils. All these alterations may modulate the charge, structure and function of the protein and thereby affect the propensity for misfolding and aggregation [29,30].

The alpha-synuclein protein was identified over 100 years ago. Nonetheless, to date, the physiological function of the protein remains unclear. Owing to its localization at the synapse, it is thought to play a central role in synaptic activity and plasticity, neurotransmitter release and synaptic vesicle trafficking [2,31–34]. Moreover, as the N-terminal domain of the protein can interact with membranes, it might play essential roles in membrane organization, in sensing and stabilizing curved membranes, and it may interact with other membrane-associated proteins or membranous organelles, including the nucleus, endoplasmic reticulum (ER) and mitochondria, as well as mitochondria-associated membranes (MAMs) [35–41]. It also seems that alpha-synuclein is involved in the maintenance of mitochondrial bioenergetics, as the protein can interact with several components of the electron transport chain (ETC), such as ATP synthase and complex I [42,43]. Although the exact function of alpha-synuclein remains to be determined, its proposed functions may indicate the origin of alpha-synuclein toxicity.

## 2. Alpha-synuclein pathobiology

While physiologically mostly soluble and unstructured, or membrane-bound through a partial α-helical secondary structure, in pathological conditions, alpha-synuclein can adopt oligomeric and fibrillar states, which have been shown in both *in vitro* [44–47] and *in vivo* studies [48–50]. These structural changes ultimately lead to the formation of inclusions, such as Lewy bodies (in the neuronal cell body), Lewy neurites (in the neuronal processes) or glial cytoplasmic inclusions (GCIs) (figure 1-Ib), which are associated with different neurodegenerative diseases collectively called synucleinopathies, including PD, multiple system atrophy (MSA) and DLB [16,51–55]. Currently, it is believed that there are two main pathways by which alpha-synuclein can form such inclusions, as depicted in figure 1. The first pathway is the conventional deposition pathway in which proteins move from their native state to the amyloid state via more ordered oligomeric and fibrillar intermediate states [56]. The other option is the condensation pathway, in which native protein converts into an amyloid state by moving through a condensed, droplet-like state that can transition into more gel- and solid-like states, eventually forming amyloids [57–64]. In both cases, the protein undergoes a transition into a more beta-sheet-rich structure to form amyloids, which eventually leads to neurodegeneration (figure 1-VIII) [59,65,66]. Furthermore, it is also thought that these two pathways are not completely separated but might be linked, or the condensation pathway even precedes the aggregation [63,67,68].

**Figure 1.**
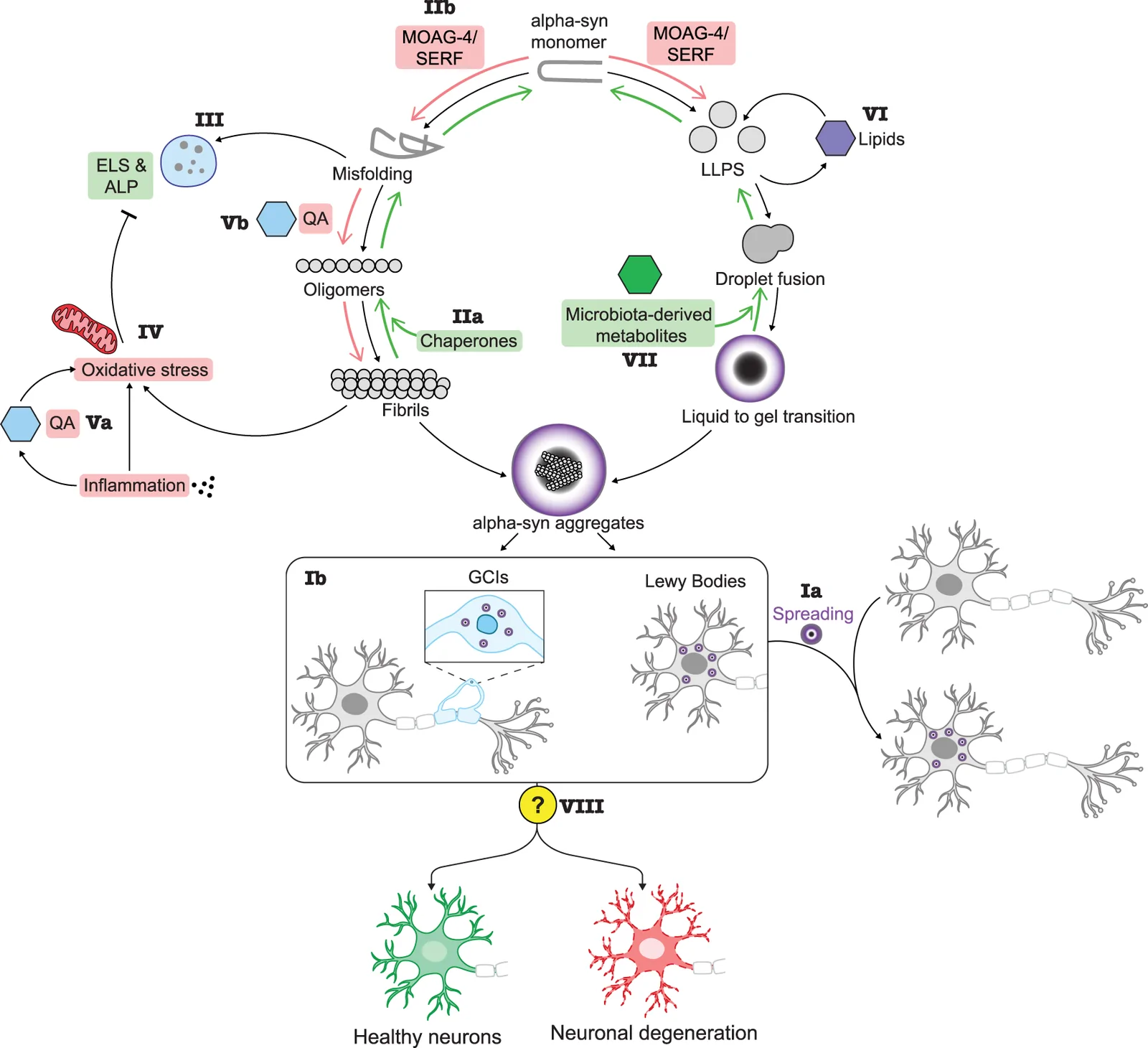
Cellular factors that influence and are influenced by alpha-synuclein toxicity. Alpha-synuclein can form aggregates through the conventional pathway with oligomer and fibril intermediates or through LLPS, in which liquid protein droplets transition into a gel-like state that matures into aggregates and (Ia) spreads from neuron-to-neuron. These aggregates can be observed inside neurons, called Lewy bodies or Lewy neurites, or in glial cells, called glial cytoplasmic inclusions (GCIs) (Ib), and can lead to neurodegeneration (VIII) through still unknown mechanisms. (IIa) Chaperones and (VII) microbiota-derived metabolites can lower the amount of aggregates or inclusions, while (IIb) modifier of aggregation 4 (MOAG-4)/small EDRK-rich factor (SERF) promotes inclusion/aggregate formation. Other cellular processes and molecules, such as (IV) oxidative stress, (Va) inflammation, (III) autophagy and the endolysosomal system (ELS) and (Vb) quinolinic acid (QA) and (VI) lipids also affect the aggregation/phase separation of alpha-synuclein.

Initially, *in vitro* studies have shown that alpha-synuclein can undergo LLPS, forming a condensed state that could transition into a more solid-like amyloid form containing oligomers and fibrillar species [59,64,69]. Subsequently, studies in mammalian cells as well as other model organisms also showed the presence of alpha-synuclein liquid condensates that could mature into solid aggregates [64]. The propensity of alpha-synuclein to phase separate or aggregate depends on the amino acid sequence (different disease-associated mutations and PTMs increase the aggregation propensity), the environmental conditions (e.g. cellular milieu, stress and oxidation) and their interactions [58,63,67,70–72]. The arginine-soluble N-ethylmaleimide-sensitive factor attachment protein receptor (R-SNARE), vesicle-associated membrane protein 2 (VAMP2) and other synaptic proteins, such as synapsins, as well as interactions with lipid membranes, can help to mediate the phase separation of alpha-synuclein [9,73–77]. Eventually, the accumulation of alpha-synuclein results in protein aggregates that can be found in different brain regions and can be associated with different diseases. Athough alpha-synuclein is the main component of these aggregates, over 90 different molecules, including lipids, cytoskeleton proteins, heat-shock proteins (HSPs), fragmented organelles, vesicles, lysosomes, mitochondria and others, have been identified in aggregates [78–84]. The composition of these aggregates may hold information about their formation and toxicity in the cell.

Historically, amyloid fibrils of alpha-synuclein were thought to be toxic, but ample research [85–96] suggests that other assemblies, such as small oligomers, may be more toxic than amyloid fibrils. It is likely that different alpha-synuclein intermediates on the pathway to fibril formation cause toxicity through different mechanisms.

Furthermore, it has been shown that alpha-synuclein can be excreted from neurons and taken up from the environment through passive diffusion as well as via active pathways, such as endocytosis, spreading in a prion-like manner and causing toxicity [53,97–101]. Another release mechanism is through association with exosomes, which are small vesicles that can be secreted from various cell types [102,103]. After secretion into the extracellular space, uptake of alpha-synuclein by other cells contributes to the seeding of endogenous protein aggregation, as well as cell-to-cell spread (figure 1-Ia). Uptake of alpha-synuclein monomers, oligomeric species or fibrils can happen passively through diffusion or actively through different means of endocytosis, such as pinocytosis and phagocytosis [101,104]. Overall, alpha-synuclein can be considered to exhibit several prion-like characteristics but does not seem to function as a transmissible prion protein.

In this review, we will outline the fundamental biological mechanisms of alpha-synuclein toxicity at the cellular, organismal and systemic levels, as well as the processes that can ameliorate alpha-synuclein toxicity, and address the remaining open questions regarding alpha-synuclein toxicity.

## 3. Modifying mechanisms at an organismal scale

### (a) Cell-intrinsic factors

#### (i) The impaired proteostasis network

The accumulation of alpha-synuclein in the different synucleinopathies results in a continuous state of stress that affects the proteostasis network [105,106]. This network consists of multiple pathways that are responsible for the synthesis, maintenance and degradation of proteins to preserve a balanced proteome [105–107]. Upon ageing, these proteostatic pathways become impaired, resulting in protein aggregation and cellular dysfunction [105–108]. This section will dive into the mechanisms by which alpha-synuclein can affect and is affected by different pathways of the proteostasis network.

#### (ii) The effect of alpha-synuclein on protein synthesis

Protein synthesis is an energy-consuming process that has been implicated in synucleinopathies. Furthermore, in combination with mitochondrial dysfunction, an increase in protein synthesis can further overwhelm the proteostasis network, which is already compromised in these diseases [105,106]. Ultimately, this leads to neuronal death.

Several proteins involved in protein synthesis, including the eukaryotic initiation factor eIF4G1, have been linked to synucleinopathies, such as PD and DLB [109–111]. This initiation factor is an important scaffold for the translation initiation complex, which recruits tRNAs and ribosomes to the mRNA in the translation process [112]. Furthermore, there is a link between *eIF4G1* and *VPS35*, another PD risk gene, that is part of the retromer complex, which mediates protein transport from the endosomes to the trans-Golgi network [113,114]. Another genetic link between protein translation and synucleinopathies comes from the leucine-rich repeat kinase 2 (*LRRK2*), another genetic risk factor for PD. This protein is a phosphatase and when mutated, it hyperphosphorylates the translation repressor protein eukaryotic translation initiation factor 4E-binding protein*,* increasing protein synthesis and resulting in oxidative stress resistance, dysregulated calcium homeostasis and neurodegeneration [109,115,116]. However, other studies report that LRRK2 suppresses protein synthesis [117], especially of mRNAs with complex 5′UTR secondary structures [118] or of regulators of proteostasis [119]. LRRK2 was also shown to interact with other protein translation initiation and elongation factors, as well as ribosomal proteins, showing the connection between PD risk genes and translation dysregulation [120,121]. Even though most current evidence showing dysregulation of protein synthesis comes from disease-linked mutations, it is likely that this process is also affected in the absence of these mutations owing to the presence of pathological alpha-synuclein species.

One indication that alpha-synuclein (indirectly) affects the overall process of protein synthesis is its interaction with the decapping module of P-bodies, thereby interfering with mRNA decay, P-body homeostasis and mRNA stability [122]. P-bodies are membraneless, cytoplasmic ribonucleoprotein granules that primarily consist of translationally repressed mRNAs or proteins related to mRNA degradation [123,124]. As alpha-synuclein can modulate P-bodies and mRNA stability, it might also lead to alterations in protein synthesis. Another way in which alpha-synuclein can affect protein translation is through the unfolded protein response (UPR), another key pathway in the proteostasis network [105,125]. Upon activation of the UPR by pathological alpha-synuclein, overall protein synthesis is reduced while the synthesis of molecular chaperones and components of the degradation pathway are enhanced [125,126]. Additionally, protein synthesis may be challenged by collision and stalling of ribosomes on transcripts, resulting in the production of aberrant protein species and the activation of ribosome quality control, as can occur during ageing [127]. This, in turn, can lead to protein species that contribute to neurodegenerative diseases. Lastly, the mechanistic target of rapamycin (mTOR), a master regulator of many cellular processes, including protein synthesis, has also been implicated in the pathogenesis of synucleinopathies [128,129]. The exact role of mTOR in pathogenesis remains unknown as there are reports of both neuroprotective and neurotoxic effects in different synucleinopathy models [128]. Recently, it was reported that alpha-synuclein causes a deregulation of mRNA translation in PD through destabilization of a cellular complex that triggers mTOR complex 1 activation, leading to increased translation and contributing to neurodegeneration [129]. However, as mTOR is a key regulator of many cellular processes, including autophagy, proliferation and metabolism, more mechanistic studies examining the effect of mTOR activity on pathogenesis are required.

#### (iii) Chaperones and alpha-synuclein

Molecular chaperones are also key players in maintaining a functional proteome because they assist in protein folding, prevent misfolding and help with protein degradation in both ATP-dependent and independent mechanisms [105,106,130]. The transcription of many chaperones is induced under stress conditions in the cell, which is often paired with general attenuation of protein synthesis so there is less production of new chaperone clients [105,130]. Furthermore, some chaperones can also act as disaggregases, which actively dissolve protein aggregates in an ATP-driven process. Ample research has shown that chaperones can interact with different alpha-synuclein species [131–134]. Additionally, it was shown that a complex of three different molecular chaperones, called HSPs—HSP110, HSP40 and HSP70—could selectively depolymerize and dissolve alpha-synuclein fibrils [133,135]. During ageing, the expression of these chaperones is repressed, contributing to the impaired proteostasis network [136].

In several model organisms, it has been shown that the (over)expression of chaperones can suppress alpha-synuclein toxicity (figure 1-IIa) [137–139]. For example, in a *Drosophila* model, the expression of HSP70 was able to protect dopaminergic neurons against alpha-synuclein-induced neurodegeneration as a consequence of alpha-synuclein toxicity [140]. Similarly, overexpression of HSP110 was shown to mitigate alpha-synuclein toxicity in cell culture as well as in two mouse models [141]. Furthermore, in a cellular model, it was shown that alpha-synuclein aggregation depends on the chaperones DnaJ heat shock protein family (Hsp40) member B6 (DNAJB6) and HSP70 [142]. Moreover, transcriptomic analysis of post-mortem brains of PD patients showed an inverse correlation between chaperone expression in excitatory neurons and alpha-synuclein pathology, suggesting that lower chaperone levels contribute to Lewy body pathology [143]. These results show the potential of modulating chaperone levels in mitigating alpha-synuclein toxicity.

While molecular chaperones are actively workin to prevent the formation of protein aggregates, there are regulators of proteostasis that actively promote the aggregation of aggregation-prone proteins. One such regulator is the *Caenorhabditis elegans* modifier of aggregation 4 (MOAG-4), homologue of small EDRK-rich factor 1A (SERF1A) and SERF2 in humans, which has been shown to drive amyloid formation of amyloid-beta, huntingtin and alpha-synuclein (figure 1-IIb) [144–146]. Furthermore, it was shown that MOAG-4/SERF drives higher-order assemblies and condensates, accelerating aggregation [68,147,148], probably through charge–charge interactions with the C-terminus of alpha-synuclein [144,147,149]. Inhibition of MOAG-4 was shown to decrease protein aggregation and toxicity in a *C. elegans* model [145].

#### (iv) Affected protein degradation and transport

Another essential part of the proteostasis network is the degradation pathways. There are two main proteolytic pathways: the ubiquitin–proteasome system (UPS) and the autophagy–-lysosomal pathway (ALP) [150]. In synucleinopathies, these pathways are often compromised, resulting in impaired clearance of alpha-synuclein [105,106,151]. For example, it has been observed that PD patients have lower levels of proteasome subunits, such as the 20S proteasome core and the 26S proteasome, which both lead to dysfunction of the UPS [152–156]. Furthermore, oligomeric or filamentous alpha-synuclein interacts with proteasome subunits, inhibiting their function and leading to impaired proteasomal degradation of damaged and misfolded proteins [157,158]. The ALP is involved in the degradation of proteins, aggregates and organelles through the lysosome via macroautophagy and chaperone-mediated autophagy (CMA). It has been shown that pathological alpha-synuclein can block lysosomal translocation, which impairs degradation [151,159].

Beyond the direct degradation pathways, the endolysosomal system (ELS) plays an important role in orchestrating intracellular trafficking, processing and delivery of proteins to the right degradation compartments as well as the uptake and processing of cellular material [160]. There is increasing genetic and clinical evidence that the ELS is impaired in neurodegenerative disorders, including synucleinopathies, often resulting in the impaired clearance of toxic alpha-synuclein species through CMA [151,159,161]. Genetic studies have shown that mutations impairing functions of lysosome-associated genes, including *glucocerebrosidase 1* (*GBA1*), *LRRK2*, *VPS3* and *ATPase cation transporting 13A2* (*ATP13A2*), are strongly associated with the risk and progression of disease [151,162–165].

Monomeric and oligomeric alpha-synuclein can interact with trafficking vesicles from the ELS, disrupting vesicle trafficking and protein degradation by impairing the ALP (figure 1-III) [104,166,167]. It was shown that alpha-synuclein colocalizes with early, recycling and late endosomes [166]. This results in a positive feedback loop in which the lysosomal degradation capacity declines and alpha-synuclein accumulates, further disrupting these organelles. Microscopy studies have shown fragmented and swollen lysosomes in patient material, as well as the presence of lysosomes in alpha-synuclein aggregates [82,168]. Furthermore, dysfunction of the ELS and the retromer complex may be involved in the spreading of disease pathology. Vesicles of endocytic origin, called exosomes [169], have been shown to contain alpha-synuclein and mediate cell-to-cell transmission and the progressive spread of disease pathology [170–172].

Owing to the observed endolysosomal dysfunction in patients suffering from synucleinopathies, considerable efforts have been made to investigate the therapeutic value of targeting the ELS. Studies have examined enhancing lysosomal biogenesis and autophagic flux to clear alpha-synuclein species from the cell. Transcription factor E-box binding (TFEB) is a master regulator of the ALP and lysosomal biogenesis, and its overexpression of TFEB can alleviate alpha-synuclein-induced toxicity and increase autophagy in several synucleinopathy models [173–175]. Additionally, one could modulate lysosomal enzyme activity to restore lysosomal function. Another strategy is targeting lysosomal vesicle trafficking to impair alpha-synuclein accumulation and propagation [176]. In synucleinopathies, there is impaired lysosomal trafficking of cathepsins in the lysosome, which are involved in alpha-synuclein degradation. By boosting the lysosomal transport of these proteins, alpha-synuclein levels can be lowered [177]. Furthermore, modulating retromer function, for example by targeting *SNX5*, was shown to improve autophagy and lysosomal enzyme recycling [165].

Current research supports the importance of dysfunction of the degradation pathways and the ELS in synucleinopathies; especially, the vicious cycle between alpha-synuclein pathology and lysosomal impairment underscores the importance of the system in disease, but it remains unclear whether this initiates synucleinopathy or is a consequence of disease. The main significant challenges include the precise molecular mechanisms, the crosstalk between organelles and possible therapeutic strategies. Even though synucleinopathies show a characteristic decrease in the efficiency of the proteostasis network, there are other cellular processes that affect alpha-synuclein toxicity and *vice versa*; alpha-synuclein toxicity probably affects different cellular pathways. These will be further discussed in the following sections.

#### (v) Altered mitochondrial functioning

Mitochondrial dysfunction and oxidative stress (figure 1-IV) have long been implicated as a disease hallmark of synucleinopathies, and several genetic mutations (in *PINK1, PRKN, LRRK2* and *DJ-1)* affecting mitochondrial functioning and dynamics are associated with these diseases [178]. Neurons are high-energy-demanding cells that require ATP production through mitochondrial oxidative phosphorylation and glycolysis to maintain ionic homeostasis for their functioning [179]. Furthermore, mitochondria play an important role in the regulation of calcium homeostasis, the generation and neutralization of reactive oxygen species (ROS), and apoptosis [180–185]. Calcium homeostasis is important for the release of synaptic vesicles, and an excess of calcium can lead to cellular damage and cell death [186]. Furthermore, mitochondria are involved in the transfer of calcium towards the ER to maintain intracellular calcium stores [187–189].

It was shown that Lewy bodies can contain dysmorphic mitochondria [82]. Additionally, the structure of pathological alpha-synuclein allows it to interact with highly curved membranes, such as those of mitochondria [190]. These interactions can disrupt the assembly of ETC complexes, (MAMs), as well as induce the fragmentation of the organelle [43,188,191–193]. Furthermore, alpha-synuclein can disturb the ER–mitochondria associations that are important for the delivery of Ca^2+^ to mitochondria for proper ATP synthesis, which in turn is important for synaptic signalling [187,189,194]. Therefore, neuronal and synaptic function are also affected by the accumulation of damaged and dysfunctional mitochondria as shown by down-regulation of synaptic and mitochondrial genes in primary neurons and in mice [97,195,196]. Additionally, alpha-synuclein leads to the impairment of the ATP synthase and mitochondrial respiration, which results in mitochondrial depolarization leading to the opening of the mitochondrial permeability transition pore (mPTP), which triggers mitochondrial swelling and cell death [190]. It was also found that the interaction of alpha-synuclein with mitochondrial membranes can preferentially trigger alpha-synuclein aggregation [197].

Even though there is a clear link between alpha-synuclein pathology and mitochondrial dysfunction, the role of alpha-synuclein in normal mitochondrial physiology, as opposed to its contribution to pathology when misfolded, remains elusive. Further elucidation is necessary to disentangle the bidirectional relationship between mitochondrial stress and alpha-synuclein aggregation, where one might promote the other and* vice versa*. Through selective manipulation of mitochondrial function in different alpha-synuclein models, further insight into the responsibility of mitochondria in neuronal dysfunction and death can be gained.

### (b) Systemic factors

#### (i) Rewiring of amino acid metabolism

Many alpha-synuclein toxicity modifiers have been discovered by exploring the mechanisms of genetic risk factors underlying PD. Alternatively, high-throughput unbiased screens have also elucidated toxicity modifiers [198]. One example is the knockdown of tryptophan 2,3-dioxygenase (TDO2), the rate-limiting enzyme of the kynurenine pathway (KP) that metabolizes tryptophan into downstream neuroactive metabolites [199,200]. Tryptophan is initially converted to N'-formylkynurenine and kynurenine, which are either metabolized further into kynurenic acid (KA) or 3-hydroxykynurenine (3-HK), which eventually forms metabolites like quinolinic acid (QA) [200]. The KP is highly evolutionarily conserved from humans and other mammals to *Drosophila* and *C. elegans* [199]. The downstream metabolites of the KP have many significant physiological roles in neuronal health. For example, QA is neurotoxic as it binds and activates N-methyl-D-aspartate (NMDA) and α-amino-3-hydroxy-5-methyl-4-isoxazolepropionic acid (AMPA) glutamate receptors in excitatory neurons, triggering excitotoxicity [200,201]. Conversely, KA is thought to be neuroprotective owing to its activity as a general antagonist to glutamate receptors, as well as an antagonist to acetylcholine receptor α7nAChR, thus further protecting against proteotoxicity [202].

The discovery of the protective effect of TDO2 loss has led to extensive studies searching for a correlation between KP metabolites and neurodegenerative disease. Multiple studies have shown significant alterations in tryptophan and metabolite levels in neurodegenerative proteinopathies, including PD and MSA [203]. PD patients displayed an increased ratio of QA/KA in both their plasma and cerebrospinal fluid [204]. Specifically in PD, a significant increase in 3-HK and QA in PD patient blood plasma and cerebrospinal fluid (CSF) [200,204,205], as well as a downregulation of KA [205,206] are observed. The significant dysregulation of KP metabolite levels in patient plasma and CSF may suggest that KP metabolites could serve as reliable biomarkers for PD [204]. A summary of alterations in KP levels in PD, DLB and MSA patients was thoroughly reviewed in Kaleta *et al.* [203].

These significant correlations between KP metabolites and synucleinopathy progression suggest a tight interplay between the KP and alpha-synuclein toxicity. Inflammation promotes the activity of indoleamine 2,3-dioxygenase (IDO, a differentially regulated functional homologue of TDO2 in mammals) [207]. This, in turn, promotes the activity of the KP, potentially increasing levels of 3-HK and QA, which further increase oxidative stress (figure 1-IV) and glutamate receptor overactivation (figure 1-Va) [208,209]. Several studies have shown that inducing oxidative damage can increase alpha-synuclein aggregate formation [210,211]. Furthermore, QA can directly enable the seeding of alpha-synuclein aggregation *in vitro* (figure 1-Vb) [212]. Finally, polymorphisms of the KP gene *ACMSD*, which may boost QA levels, have been found to be associated with PD in several genome-wide association studies [213,214]; however, this correlation appears to be population-dependent [215,216]. Moreover, a recent study in zebrafish found that while *ACMSD* deficiency does boost QA levels, no significant pathologies were identified, probably owing to protective changes in the KA : kynurenine ratio that were also induced upon loss of *ACMSD* [217]. Overall, evidence suggests that there is a positive-feedback loop between the balance of neurotoxic and neuroprotective KP metabolites, alpha-synuclein, inflammation and oxidative stress, that may ultimately exacerbate alpha-synuclein toxicity.

An increasing body of evidence points towards a systemic neuroprotective effect of losing TDO2. There has been extensive research into the link between tryptophan metabolism and the KP and neuroprotection in models of PD as well as other models of neurodegenerative diseases, including Alzheimer's disease (AD) [199,200,205,218,219]. TDO2 therefore makes an attractive therapeutic target owing to its broad species-wide protection against proteotoxicity. In a mouse AD model, oral administration of a TDO2 inhibitor improved memory, but had no impact on blood or brain tryptophan metabolite levels [218]. In a separate study in a mouse PD model, two separate TDO2 inhibitors alleviated both motor and cognitive dysfunction, but only one inhibitor boosted blood and brain tryptophan levels, while the other did not [219]. Overall, these studies suggest that restricting **TDO2 activity does not consistently translate to a change in tryptophan levels in the brain. Therefore, the mechanism that mediates protection upon TDO2 depletion is probably mediated by a cell non-autonomous mechanism that enhances neuronal resilience against proteotoxicity and ageing. This effect may act via the systemic circulation of a KP metabolite capable of crossing the blood–brain barrier (BBB), or via a KP-independent mechanism.

Overall, tryptophan metabolism may represent a key metabolic switch that contributes to systemic protein homeostasis and protects against protein toxicity. As an essential amino acid, tryptophan availability may directly influence translation, therefore we hypothesize that KP perturbations may have a systemic effect on protein synthesis. For example, elevated TDO2 or IDO) levels may lower tryptophan levels and potentially impair translational fidelity, thereby increasing the risk of protein misfolding. In addition, KP metabolite QA can directly modulate alpha-synuclein aggregation *in vitro*, which may indicate a more direct link between tryptophan metabolism and alpha-synuclein toxicity [212]. However, despite extensive research into the mechanisms of neuroprotection via KP interference in PD and other neurodegenerative disease models, the mechanisms of neuroprotection are still poorly understood. Further understanding of this mechanism may be instrumental in the discovery of pharmacological interventions against synucleinopathies and other diseases of the nervous system.

#### (ii) Altered lipid metabolism

Converging lines of evidence frame lipid homeostasis as a central determinant of alpha-synuclein pathobiology, as meticulously reviewed in [220–222]. In addition to genetic associations with lipid-related loci, such as *GBA1, ELOVL7, DGKQ, SREBF1, PLA2G6, VPS13C, CRLS1, FASN1, SYNJ1* and *SNCA* in PD [223–233]; *TMEM175* and *APOE* in DLB [226,234–237]; and more controversially, *ELOVL7* and *COQ2* in MSA [238–241], complementary lipidomic analyses of brain tissue, biofluids and disease models consistently show shifts toward altered fatty acid chain-length and unsaturation in alpha-synuclein pathology [242–244]. Moreover, ultrastructural analyses of Lewy bodies in the brains of PD patients have revealed a dense clustering of membranous, lipid-rich structures, some including vesicles coated with alpha-synuclein [82,84]. Notably, these studies found that fibrillar assemblies of alpha-synuclein were apparent in less than 20% of Lewy bodies, supporting a broader conceptual framework that redefines synucleinopathies as both proteinopathies and lipidopathies.

Extensive research has shown that long-chain unsaturated fatty acids can promote alpha-synuclein aggregation and cytotoxicity *in vitro* (figure 1-VI), whereas saturated fatty acids act as negative modulators of aggregation [40,245–247]. On aggregation dynamics, Galvagnion *et al.* [248] showed that lipid vesicles trigger alpha-synuclein aggregation via primary nucleation. In the cellular context, lipids with higher ratios of long, unsaturated fatty acids may introduce packing defects in membranes, which favour both interaction with alpha-synuclein and the crowding of membranes with alpha-synuclein monomers [249,250]. This would partly explain the lower alpha-synuclein tetramer-to-monomer ratio observed in diverse PD models [11,251–254] and could establish an important foundation to better understanding cell-autonomous aspects of alpha-synuclein pathobiology, such as defects in endolysosomal trafficking and mitochondrial dysfunction (as mentioned in §§3a(iv,v)).

However, given that lipids constitute a highly diverse chemical space and are present in cells as structural components of membranes, scaffolds for protein localization, energy storage molecules, signalling mediators and metabolic intermediates [255], it stands to reason that lipid levels at the cellular and systemic levels (i) reflect the risk, presence or progression of synucleinopathies, and (ii) hold potential to modify disease onset and progression.

Consistent with this view, neuron–astrocyte lipid coupling provides a crucial non-cell-autonomous buffer: during heightened activity, neurons export excess fatty acids that astrocytes sequester in lipid droplets and catabolize via β-oxidation, thereby limiting neuronal lipotoxicity [256]. With ageing and vulnerability states, this buffering appears to weaken or become dysregulated, as suggested by work showing that stressed/ageing glia accumulate lipid droplets and by genetic risk contexts that impair neuron–glia fatty acid handling [257–260]. In PD, post-mortem analyses report increased neutral lipids in dopaminergic neurons and microglia coupled to decreased neutral lipids in astrocytes from the same subjects, pointing to impaired intercellular lipid homeostasis [261].

Building on the neuron–glia intercellular insights, multiple lipidomic analyses of patient-derived brains and biofluids reveal that changes in lipid composition also manifest at a systemic level in synucleinopathies. In PD, patient brains exhibit reduced triacylglycerol (TAG) in the primary visual cortex [262], elevated TAG in the motor cortex [263] and increased TAG and diacylglycerol (DAG) in the motor [263] and frontal cortices [264]. However, the brain lipidome landscape is far more complex, as region-specific remodelling in PD spans the abundance, chain-length and degree of unsaturation of many more lipid classes, such as increased cholesteryl esters, unsaturated phosphatidylserine and unsaturated phosphatidylcholine (PC) in the substantia nigra pars compacta [265], along decreased unsaturated phosphatidylethanolamine (PE) and chain-length dependent changes of phosphatidylinositol in the visual cortex [262].

Complementarily, serum [266–268] and CSF [266] studies demonstrate broad shifts in PD lipid profiles: circulating levels of sphingolipids and glycerolipids are significantly perturbed in PD patients (including those with *LRRK2* mutations), particularly ceramides, phosphatidylcholines, lysophosphatidylethanolamines, acylcarnitines, sphingomyelins and TAG [266,267]. Generally, serum TAG levels are lower in PD patients [268–270] and associated with more severe motor symptoms [268], while metabolic syndrome but not high serum TAG (i.e. hypertriglyceridemia) have been associated with a higher risk of PD [271]. Changes towards lower circulating TAG levels in PD might reflect the observed accumulation in specific brain regions and indicate a systemic lipid metabolism dysfunction.

In DLB, lipid deficits are most consistently reported as reductions in phospholipids, particularly phosphatidylinositol-4,5-bisphosphate (PIP2), with stronger signals among APOE-ε4 carriers in AD-context cohorts [235,272], as well as reduced DAG in the frontal cortex [264]. Notably, APOE-ε4 carriers exhibit exacerbated amyloid-independent alpha-synuclein pathology [273]. Finally, in MSA, systemic lipid profiles typically show reduced levels of cholesterol, low-density lipoprotein cholesterol (LDL-C), high-density lipoprotein cholesterol (HDL-C) (especially in multiple system atrophy, cerebellar type (MSA-C)) and triglycerides, independent of disease progression [274–276]. Additionally, lipidomic analysis of motor cortex white matter revealed elevated levels of phospholipids enriched in monounsaturated fatty acids, particularly phosphatidic acid, PE and PC [277].

Until this point, it has been established that lipid metabolism can modulate proteostasis through a remodelling of glycerolipids, phospholipids and sphingolipids that tunes membrane charge, curvature and packing—all key determinants of alpha-synuclein conformation, condensation and nucleation. The shifts in neutral lipids and phospholipids towards longer-chain and more unsaturated fatty acid composition observed in synucleinopathies are consistent with the pro-aggregation environment described *in vitro*, altering membrane binding, phase behaviour and fibrillation kinetics [243,246,248]. Another implication emerges in the proteostasis–lipid axis, where GBA1-linked glucosylceramide/ceramide/sphingomyelin imbalance stabilizes toxic oligomers and compromises lysosomal lipid handling, reinforcing alpha-synuclein accumulation and spread [278–280]. Collectively, lipid dyshomeostasis also impairs autophagy–lysosome and proteasome function via disrupted PIP2 signalling and membrane trafficking, weakening proteostasis and amplifying toxicity across synucleinopathies [278,281–284].

Together, these multi-tissue lipidomic alterations reinforce a model wherein lipid dysregulation and alpha-synuclein pathology are intimately connected and mutually reinforcing. Aberrant lipid compositions (e.g. loss of critical membrane lipids or accumulation of oxidized/unsaturated species) can promote alpha-synuclein misfolding and aggregation and impair clearance, while conversely alpha-synuclein aggregates can secondarily perturb cellular lipid homeostasis, establishing a feed-forward cycle driving neurodegeneration in PD, DLB and MSA.

As our understanding deepens, lipid-centred interventions (from fatty acid modifiers and lipid metabolism modulators to dietary interventions) are emerging as promising disease-modifying therapies that maintain the complex balance between alpha-synuclein and the lipid-rich milieu of the brain, and of cells themselves.

### (c) Environmental factors

#### (i) Diet, microbiome, infections and toxins

Alpha-synuclein can affect and be affected by not only cell-intrinsic and systemic pathways, but also by cell-extrinsic factors that play a big part in modifying toxicity (figure 2). These include diet, microbiome, infections, inflammation and toxins.

**Figure 2.**
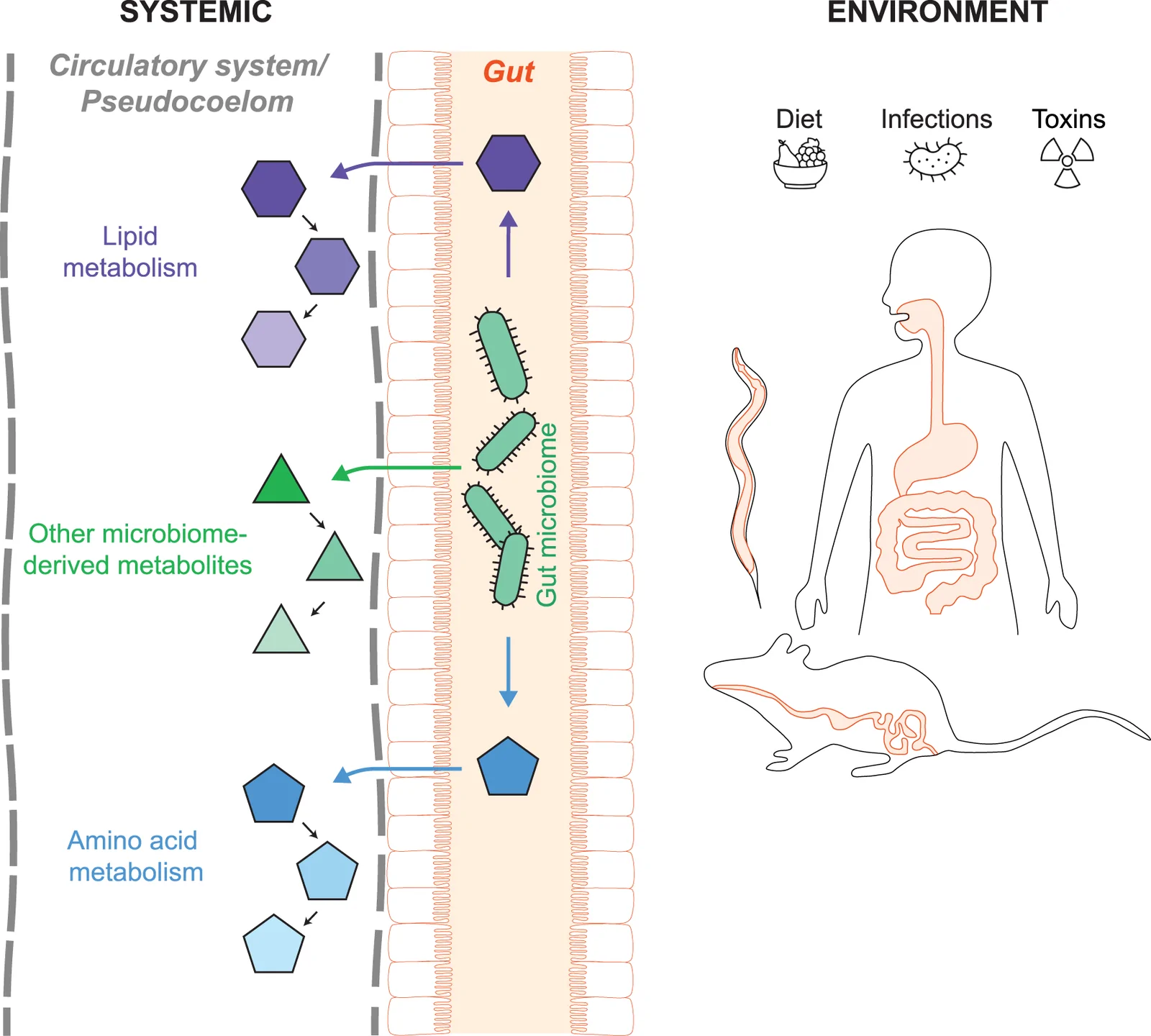
Systemic and environmental factors that influence alpha-synuclein toxicity. Different environmental factors can affect synucleinopathies; these include diet and infections, as well as toxins. These, in turn, can have systemic effects that influence disease pathology. The gut microbiome plays an important part not only in the uptake of certain metabolites from our food, but also in amino acid and lipid metabolism. These processes are also affected in synucleinopathies.

Diet and nutrient availability are strong modifiers of alpha-synuclein toxicity, controlled mechanistically either via the microbiome, amino acid metabolism or by restricted calorie intake that promotes aberrant protein clearing. Dietary interventions have shown potential in modulating alpha-synuclein pathology and neuroinflammation. A fibre-rich prebiotic diet improved motor symptoms and reduced alpha-synuclein aggregation in a mouse model overexpressing alpha-synuclein by shifting microglia from a pathological to a physiological state, while microglia depletion abolished this protective effect [285]. Additionally, modulation of the KP, which metabolizes tryptophan, may help reduce alpha-synuclein toxicity [286]. However, excessive tryptophan or high-protein intake can activate liver TDO2, lowering systemic tryptophan and increasing toxic metabolites, such as QA, while competition among amino acids limits brain uptake [287,288]. By contrast, low-protein, high-carbohydrate diets enhance brain tryptophan availability through insulin-mediated amino acid regulation [289]. Moreover, dietary timing influences alpha-synuclein pathology; intermittent fasting and caloric restriction promote autophagy and proteostasis, reduce phosphorylated alpha-synuclein and improve motor function in PD models [290–292].

Recent research also highlights the crucial role of the gut microbiome in PD pathology, showing that PD patients exhibit altered microbial compositions compared with healthy individuals, correlating with disease symptoms [293–297]. Specifically, PD is associated with a reduction in short-chain fatty acid (SCFA)-producing bacteria, such as those generating butyrate, which are vital for intestinal barrier integrity, immune regulation and neuronal protection; their depletion contributes to gut permeability and inflammation [297–300]. A reduction in SCFA levels can impact mitochondrial functioning, energy metabolism and dynamics, processes known to be affected in synucleinopathies, and increasing SCFA levels can alleviate mitochondrial stress and damage [301–303]. Additionally, microbiota-derived metabolites, including SCFAs, are important for maintaining the structure of the BBB, and upon reduction of these metabolites, the BBB becomes more permeable, resulting in neuroinflammation [304]. Fecal microbiota transplantation from PD patients worsens pathology in mice, while transplantation from healthy donors or probiotic treatments can restore microbial balance and improve PD-related symptoms [305–309]. Furthermore, bacterial metabolites from *Bacillus subtilis* have been shown to be able to inhibit and reverse inclusion formation in a *C. elegans* model for PD [310,311]. Therefore, some microbiota-derived metabolites could have a positive influence on alpha-synuclein toxicity (figure 1-VII). On the other hand, other microbial-derived molecules, including the bacterial amyloid curli, may contribute to neurodegeneration [312–314]. Communication between the gut and brain occurs through multiple pathways, including the vagus nerve, immune signalling and microbial metabolites [315,316], with the gut microbiota also influencing neurotransmitter production, such as dopamine and serotonin [317]. Alterations in tryptophan metabolism—a process modulated by gut microbes—further affect brain function and may contribute to synucleinopathy pathology [287,318]. Even though abundant research can link the gut microbiome and its metabolites to synucleinopathies, it remains uncertain whether microbiome changes are a cause or consequence of disease progression.

A common feature of neurodegenerative diseases is neuroinflammation, often triggered by the accumulation of alpha-synuclein, which activates microglia, the adaptive immune response and overall inflammatory processes [319,320]. Microglia, the resident immune cells of the brain, recognize alpha-synuclein via Toll-like receptors, initiating a pro-inflammatory signalling cascade and promoting astrocyte activation, which is crucial for synaptic and BBB maintenance [321–326]. Microglia can internalize extracellular alpha-synuclein released by neurons, yet the resulting ROS burden may exacerbate neuronal dysfunction, especially in cells with mitochondrial impairment [100,103,321,327–331]. Peripheral inflammation originating from intestinal dysbiosis may also contribute to disease onset [332]. Previously, several infectious diseases have been linked to neurodegeneration, such as the Spanish flu and PD [333]. More recent evidence indicates that brain-penetrant viral infections, including SARS-CoV-2, can promote alpha-synuclein aggregation and potentially trigger synucleinopathies [334–340]. Altogether, chronic inflammation—a hallmark of synucleinopathies—leads to a vicious cycle of neuronal loss and further neuroinflammation [341].

Furthermore, it was reported that several environmental toxins can also result in the development of neurodegenerative diseases, including synucleinopathies [342]. The neurotoxin, 1-methyl-4-phenyl1,2,3,6-tetrahydropyridine (mPTP), specifically damages the dopaminergic neurons in the substantia nigra by entering neurons through dopamine transporters and inhibiting mitochondrial complex I, depleting ATP and inducing oxidative stress, resulting in parkinsonism [343–346]. Epidemiological studies also link farming and pesticide exposure—especially to rotenone and paraquat, which are also used to create animal models of synucleinopathies—to a higher risk of parkinsonism [347]. Notably, several pesticides have been shown to be directly toxic to dopaminergic neurons [348]. The mechanisms underlying pesticide-related PD risk have been recently reviewed [346].

## 4. Conclusion and future outlook

Taken together, in synucleinopathies, many different cellular processes are affected by alpha-synuclein and* vice versa*. Many environmental, organismal and cellular processes probably affect alpha-synuclein toxicity. These processes together shed light on disease aetiology as well as giving directions to possible developments of therapeutics. However, with respect to some of the cellular changes related to alpha-synuclein pathology, it is not known whether disruptions are a cause or a consequence of disease. Are the discussed disruptions in proteostasis, the ELS, lipid metabolic changes, mitochondrial function and amino acid metabolism the cause of alpha-synuclein aggregation, or does pathological alpha-synuclein cause these disruptions? It is very likely that some or many of these processes are interconnected and create a vicious cycle of detrimental effects. Nonetheless, it remains unclear what exactly initiates the disease. Furthermore, it remains unknown what the exact roles of different alpha-synuclein assemblies, including alpha-synuclein inclusions and Lewy bodies, are and how they affect toxicity. How does alpha-synuclein lead to neuronal death? What role do the different cellular protein homeostasis mechanisms and organelles play in mitigating or aggravating disease pathology, and how can we therapeutically target these pathways to delay or prevent disease? What drives the observed heterogeneity of the synucleinopathies? And finally, how do external factors, such as diet, microbiome, immune system and toxins, directly and indirectly converge on cellular disease mechanisms? Addressing these questions will be instrumental for elucidating the mechanisms of alpha-synuclein toxicity on both cellular and physiological levels, and for enabling the translation of these mechanisms into therapeutic strategies aimed at treating, delaying or even preventing the onset of synucleinopathies.

## Data Availability

Part of figure 1 was created using BioRender (https://biorender.com). This article has no additional data.

## Theme

One contribution of 20 to a Theo Murphy meeting issue ‘ProteostaSys: a systems view of proteostasis’.
